# Construction and validation of prognostic nomograms for elderly patients with metastatic non‐small cell lung cancer

**DOI:** 10.1111/crj.13491

**Published:** 2022-05-05

**Authors:** Haishuang Sun, Min Liu, Xiaoyan Yang, Yanhong Ren, Huaping Dai, Chen Wang

**Affiliations:** ^1^ Department of Respiratory Medicine The First Hospital of Jilin University Changchun China; ^2^ Department of Pulmonary and Critical Care Medicine China‐Japan Friendship Hospital; National Center for Respiratory Medicine; Institute of Respiratory Medicine, Chinese Academy of Medical Sciences; National Clinical Research Center for Respiratory Diseases Beijing China; ^3^ Chinese Academy of Medical Sciences and Peking Union Medical College Beijing China; ^4^ Department of Radiology China‐Japan Friendship Hospital Beijing China; ^5^ Capital Medical University Beijing China

**Keywords:** elderly patients, metastasis, nomogram, non‐small cell lung cancer (NSCLC), prognostic model, SEER database

## Abstract

**Background:**

Metastatic non‐small cell lung cancer (NSCLC) is mostly seen in older patients and is associated with poor prognosis. There is no reliable method to predict the prognosis of elderly patients (≥60 years old) with metastatic NSCLC. The aim of our study was to develop and validate nomograms which accurately predict survival in this group of patients.

**Methods:**

NSCLC patients diagnosed between 2010 and 2015 were all identified from the Surveillance, Epidemiology, and End Results (SEER) database. Nomograms were constructed by significant clinicopathological variables (*p* < 0.05) selected in multivariate Cox analysis regression.

**Results:**

A total of 9584 patients met the inclusion criteria and were randomly allocated in the training (*n* = 6712) and validation (*n* = 2872) cohorts. In training cohort, independent prognostic factors included age, gender, race, grade, tumor site, pathology, T stage, N stage, radiotherapy, surgery, chemotherapy, and metastatic site (*p* < 0.05) for lung cancer‐specific survival (LCSS) and overall survival (OS) were identified by the Cox regression. Nomograms for predicting 1‐, 2‐, and 3‐years LCSS and OS were established and showed excellent predictive performance with a higher C‐index than that of the 7th TNM staging system (LCSS: training cohort: 0.712 vs. 0.534; *p* < 0.001; validation cohort: 0.707 vs. 0.528; *p* < 0.001; OS: training cohort: 0.713 vs. 0.531; *p* < 0.001; validation cohort: 0.710 vs. 0.528; *p* < 0.001). The calibration plots showed good consistency from the predicted to actual survival probabilities both in training cohort and validation cohort. Moreover, the decision curve analysis (DCA) achieved better net clinical benefit compared with TNM staging models.

**Conclusions:**

We established and validated novel nomograms for predicting LCSS and OS in elderly patients with metastatic NSCLC with desirable discrimination and calibration ability. These nomograms could provide personalized risk assessment for these patients and assist in clinical decision.

## Graphical abstract

A total of 9,584 patients were used for the construction of the nomograms according to the inclusion criteria and the model demonstrated excellent predictive performance.

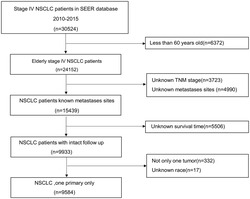

## 1 INTRODUCTION

Lung cancer is the most widespread type of cancer and the leading cause of cancer‐related deaths worldwide.^1^ Non‐small cell lung cancer (NSCLC) accounts for about 85% of all lung cancer cases, mainly including squamous cell carcinoma and adenocarcinoma subtypes. More than 1 million deaths are reported annually.^2^, ^3^ Approximately two‐thirds of NSCLC patients have local or distant metastases at the time of diagnosis, which is associated with poor prognosis. Only about 15% of these patients survive more than 5 years after diagnosis. Local metastatic sites are most commonly found in the lymph nodes (LNS) and contralateral lungs, and distant metastases often occur in the liver, brain and bone.^4^ An increasing number of patients with advanced NSCLC are over the age of 70 years,^5^, ^6^ and the proportion is increasing. The two major known oncogenic drivers in NSCLC are epidermal growth factor receptor (EGFR) mutations and anaplastic lymphoma kinase (ALK) fusions. Nonetheless, there are fewer investigations concerning the distribution of genetic mutations over different ages. Ueno et al. first prospectively assessed the role of age in EGFR mutations in 1262 patients with lung cancer and demonstrated that only 30% of patients carrying EGFR mutations were under 45 years, compared with 70% over 65 years age.^7^ It is interesting that ALK fusions were predominantly seen in in younger patients with NSCLC.^8^, ^9^ Investigating the mechanisms of age differences in the onset of different mutation types may help in the screening of the characteristic population. With the development of the aging population, the incidence and social burden of this disease will grow markedly, posing unique challenges to treatment plans. Furthermore, elderly cancer patients, including those of lung cancer, are significantly underrepresented in clinical trials and may not receive adequate treatment.^5^ Previous clinical studies have demonstrated that vinorelbine monotherapy prolongs overall survival (OS) in elderly patients with advanced NSCLC, suggesting that systemic chemotherapy may be useful in this population.^10^ Recently, treatment with carboplatin plus pemetrexed followed by maintenance treatment with pemetrexed in advanced non‐squamous NSCLC patients aged ≥75 years showed no inferiority to docetaxel monotherapy.^11^ Comprehensive geriatric assessment (CGA) is a term coined by geriatricians to describe a comprehensive assessment of functional status, co‐morbid medical conditions, cognition, psychological status, social support, nutritional status, possible geriatric syndromes, and pharmacological therapy in older individuals.^12^ The prognostic assessment based on CGA in elderly cancer patients focuses on the impact of the patient's general status and health care on patient survival. Study by Corre et al. divided elderly patients with advanced NSCLC into three groups based on the treatment of CGA, but the grouping failed to improve the survival of the patients.^13^ As such, there is still a lack of effective method to predict the survival of elderly metastatic lung cancer.

Due to limited research on the behavioral patterns of elderly patients with metastatic NSCLC and few relevant survival analyses, there is an urgent need to develop a simpler and more sensitive assessment model to individualize the prediction of this population. As a prognostic method, the nomogram contains important clinical and pathological risk factors, and can visualize the results by quantifying the impact of these variables on individual survival prediction.^14^ This method has been applied to predict the prognosis of breast cancer, bladder cancer and other cancers.^15^, ^16^, ^17^, ^18^, ^19^ To our knowledge, nomograms are not currently used to analyze the survival outcomes of elderly patients with metastatic NSCLC. Therefore, the aim of our research was to establish comprehensive nomograms to assess the prognosis of NSCLC by extracting relevant information from the Surveillance, Epidemiology and End Results (SEER) database and performed individualized survival prediction so as to provide accurate basis for clinical decision making.

## 2 MATERIALS AND METHODS

### 2.1 Study cohort

The data analyzed in the study were obtained from the SEER database, which covered almost 30% of the entire U.S. population. SEER*Stat 8.3.5 software was performed (http://seer.cancer.gov/SEERSTAT/) to access the database. Because metastatic site codes were available from 2010 in the SEER database, patients diagnosed with NSCLC between 2010 and 2015 were enrolled in this research only. The inclusion criteria were as follows: (1) histological codes were NSCLC: AD (histologic codes 8244, 8245, 8250–8255, 8260, 8290, 8310, 8323, 8333, 8480, 8481, 8490, 8507, 8550, 8570, 8571, 8574, and 8576), SQCC (histologic codes 8052, 8070–8075, 8083, 8084, 8123), large cell carcinoma (histologic codes 8012–8014), and code (8046, 8050, 8003, 8004, 8022, 8031–8035, 8082, 8200, 8240, 8249, 8430, 8560, 8562, 8980); (2) the 7th American Joint Committee on Cancer (AJCC) Stage IV patients. Patients excluded from our study were as follows: (1) patients age <60 years old; (2) the unknown TNM stage; (3) the unknown distant metastasis information; (4) lack of survival information; (5) patients with multiple primary sites. Endpoints included lung cancer‐specific survival (LCSS) and overall survival (OS). LCSS was the survival time from the date of diagnosis to a specific cancer‐related death. And OS was the time from diagnosis to death from all causes or the last follow‐up.

### 2.2 Construction and validation of nomograms

The eligible patients were randomly distributed to the training cohort (*n* = 6712) and the validation cohort (*n* = 2872) in a 7:3 ratio by applying the ‘createDataPartition’ function in the ‘caret’ package in R. In the training cohort, univariate prognostic factors with *p* < 0.05 were further incorporated into multivariate analyses. Next, prognostic factors with *p* < 0.05 in multivariate Cox regression analysis were applied to construct nomograms to predict survival outcomes (LCSS and OS).

Training set (bootstrapping method used 1000 resamples) and validation set were applied to evaluate the predictive performance of the models. The discriminability of the model was assessed by calculating the Harrell's concordance index (C‐index) with a 95% confidence interval (CI). Calibration curves was applied to compare the predicted probabilities between actual survival and the nomograms. Eventually, a decision curve analysis (DCA) was performed to evaluate the net benefit and potential clinical utility based on threshold probability. The threshold probability was used to obtain the net benefit (defined as the proportion of true positives minus the proportion of false positives, weighted by the relative harm of false‐negative and false‐positive results).

### 2.3 Comparison of nomograms

The ability of the model based on the 7th TNM staging and the nomograms established in our research was compared in the training and validation cohorts with the use of C‐index and DCAs.

### 2.4 Statistical analyses

Differences between groups were assessed by chi‐square test. Kaplan–Meier method was used for survival analysis, and differences between curves were tested by log‐rank test. Risk factors of OS and LCSS were determined by univariate and multivariate cox regression models. All statistical analyses were performed with SPSS statistical analysis software (version 24.0, IBM Corporation, Armonk, NY, USA) and R (version 3.6.0, R Foundation for statistical computing, Vienna, Austria); *p* values were bilateral, and the result with *p* < 0.05 was defined as a statistically significant.

## 3 RESULT

### 3.1 Clinicopathological characteristics

**FIGURE 1:**
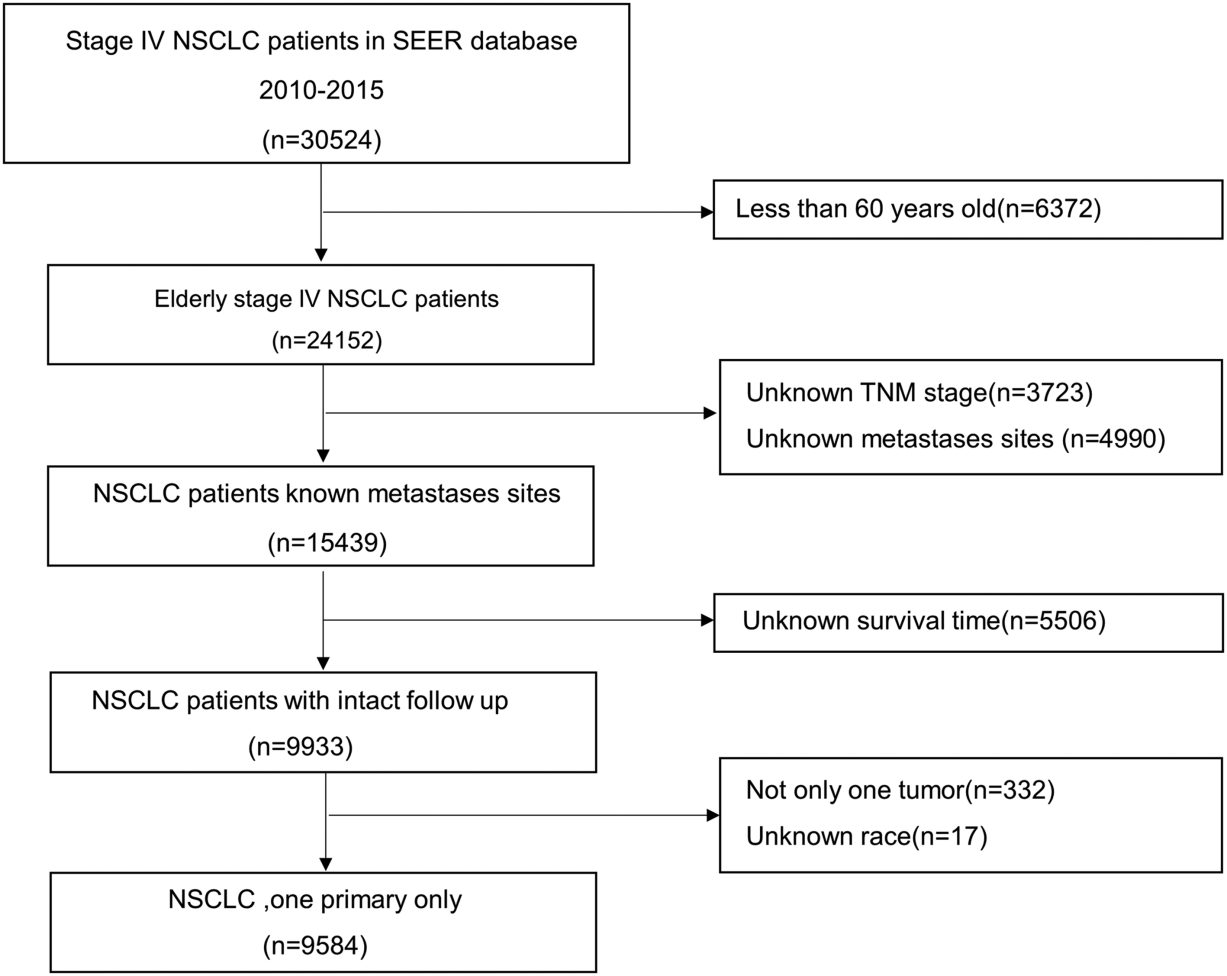
The flow diagram of eligible patients in this research

A total of 9584 elderly patients with metastatic NSCLC from SEER were eventually enrolled in our research (Figure 1). Patients were divided into different groups by the random split sample method at a ratio 7:3, of which 6712 patients in the training group and another 2872 patients constituted the validation group. The median age of the total population was 71 years (interquartile range [IQR], 66–77). In the primary cohort, the patients were mostly male (60.3%), squamous cell carcinoma (55.0%), white (80.2%), upper lung (53.1%), grade III (33.6%), T4 (36.6%), N2 (46.9%) and (multiorgan metastases, MOM) (31.2%). Meanwhile, patients were more inclined to receive less radiotherapy (10.2%) and surgery (4.0%). Detailed demographic data and clinicopathological characteristics were presented in Table 1. There was no selection bias of variables between the two groups (training and validation sets).

**TABLE 1:** Clinicopathological characteristics of patients in this research

Characteristics	Total cohort	Training cohort	Validation cohort	*p* value
	*N* = 9584 (%)	*N* = 6712 (70%)	*N* = 2872 (30%)	
**Age**				0.111
60–69	4220 (44.0%)	3002 (44.7%)	1218 (42.4%)	
70–79	3711 (38.7%)	2565 (38.2%)	1146 (39.9%)	
≥80	1653 (17.2%)	1145 (17.1%)	508 (17.7%)	
**Sex**				0.732
Male	5778 (60.3%)	4039 (60.2%)	1739 (60.6%)	
Female	3806 (39.7%)	2673 (39.8%)	1133 (39.4%)	
**Race**				0.764
White	7685 (80.2%)	5369 (80.0%)	2316 (80.6%)	
Black	1217 (12.7%)	860 (12.8%)	357 (12.4%)	
Other	682 (7.1%)	483 (7.2%)	199 (6.9%)	
**Marital status**				0.114
Married	4994 (52.1%)	3458 (51.5%)	1536 (53.5%)	
Unmarried	4194 (43.8%)	2983 (44.4%)	1211 (42.2%)	
Unknown	39 (6%)	271 (4.0%)	125 (4.4%)	
**Pathology**				0.889
Adenocarcinoma	998 (10.4%)	705 (10.5%)	293 (10.2%)	
Squamous cell carcinoma	5267 (55.0%)	3689 (55.0%)	1578 (54.9%)	
Others	3319 (34.6%)	2318 (34.5%)	1001 (34.9%)	
**Laterality**				0.501
Right	5463 (57.0%)	3811 (56.8%)	1652 (57.5%)	
Left	4121 (43.0%)	2901 (43.2%)	1220 (42.5%)	
**Primary location**				0.770
Upper lobe, lung	5088 (53.1)	3584 (53.4%)	1504 (52.4%)	
Middle lobe, lung	369 (3.9%)	251 (3.7%)	118 (4.1%)	
Lower lobe, lung	2383 (29.6)	1966 (29.3%)	872 (30.4%)	
Main bronchus	505 (5.3)	359 (5.3%)	146 (5.1%)	
Overlapping lesion of lung	96 (1.0)	70 (1.0%)	26 (0.9%)	
Lung, NOS	688 (7.2)	482 (7.2%)	206 (7.2%)	
**Grade**				0.001
I	205 (2.1%)	143 (2.1%)	62 (2.2%)	
II	1333 (13.9%)	942 (14.0%)	391 (13.6%)	
III	3222 (33.6%)	2169 (32.3%)	1053 (36.7%)	
IV	204 (2.1%)	140 (2.1%)	64 (2.2%)	
Unknown	4620 (48.2%)	3318 (49.4%)	1302 (45.3%)	
**T stage**				0917
T1	809 (8.4%)	570 (8.5%)	239 (8.3%)	
T2	2553 (26.6%)	1791 (26.7%)	762 (26.5%)	
T3	2711 (28.3%)	1907 (28.4%)	804 (28.0%)	
T4	3511 (36.6%)	2444 (36.4%)	1067 (37.2%)	
**N stage**				0.510
N0	2405 (25.1%)	1698 (25.3%)	707 (24.6%)	
N1	910 (9.5%)	623 (9.3%)	287 (10.0%)	
N2	4499 (46.9%)	3136 (46.7%)	1363 (47.5%)	
N3	1770 (18.5%)	1255 (18.7%)	515 (17.9%)	
**Surgery**				0.993
Yes	384 (4.0%)	269 (4.0%)	115 (4.0%)	
No/unknown	9200 (96.0%)	6443 (96.0%)	2757 (96.0%)	
**Radiation**				0.599
Yes	974 (10.2%)	675 (10.1%)	299 (10.4%)	
No	8610 (89.8%)	6037 (89.9%)	2573 (89.6%)	
**Chemotherapy**				0.541
Yes	5158 (53.8%)	3626 (54.0%)	1532 (53.3%)	
No/unknown	4426 (46.2%)	3086 (46.0%)	1340 (46.7%)	
**Metastasis site**				0.975
Bone	2274 (23.7%)	1601 (23.9%)	673 (23.4%)	
Brain	1436 (15.0%)	998 (14.9%)	438 (15.3%)	
Liver	720 (7.5%)	500 (7.4%)	220 (7.7%)	
Lung	2168 (22.6%)	1518 (22.6%)	650 (22.6%)	
Multiple	2986 (31.2%)	2095 (31.2%)	981 (31.0%)	

### 3.2 Survival outcomes with different metastasis sites

**FIGURE 2:**
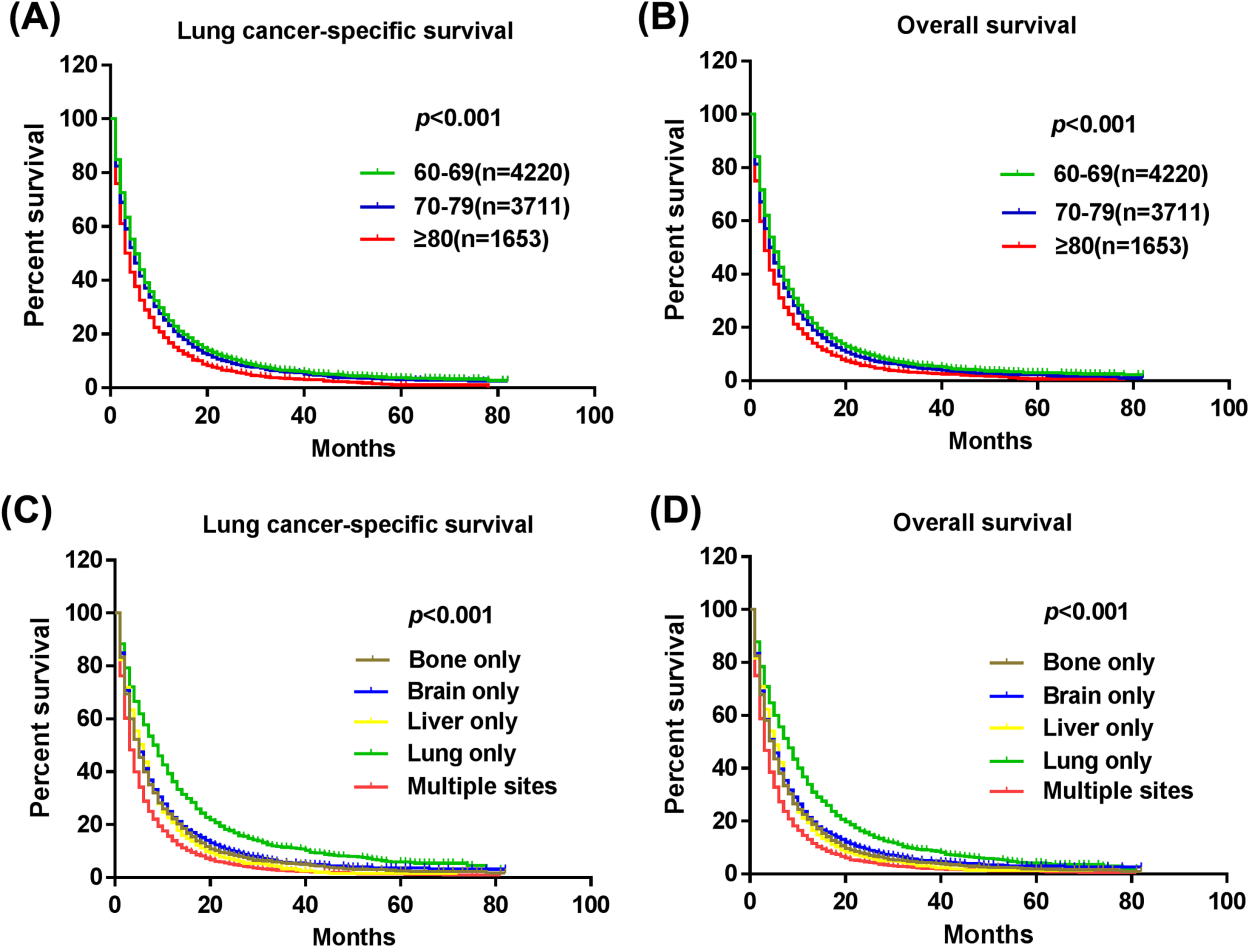
Kaplan–Meier curve of elderly patients with metastatic NSCLC. (A) LCSS (*p* < 0.001) and (B) OS (*p* < 0.001) according to age at diagnosis. (C) LCSS (*p* < 0.001) and (D) OS (*p* < 0.001) according to metastasis sites. LCSS, lung cancer‐specific survival; OS, overall survival

Among the total population, the median survival time was 5 (IQR, 2–11) months. First, we conducted survival analysis on patients of different ages, and discovered that patients with poor prognosis were mainly concentrated in patients with a diagnosis age ≥80 years (Figure 2A,B). With the analysis of different metastatic sites, we discovered that patients with multiple organ metastases had the worst 1‐, 2‐, and 3‐year survival rates (LCSS: 14%, 5.2%, 2.5%; OS: 13%, 4.6%, 2.3%), followed by patients with liver metastases alone (LCSS: 20.8%, 7.1%, 3.3%; OS: 19.3%, 6.5%, 3.1%). Nevertheless, patients with lung metastases only had better 1‐, 2‐, and 3‐year survival rates compared with other metastatic sites (LCSS: 36.3%,17.7%,11.3%; OS: 33.8%, 15.7%, 9.3%) (Figure 2C,D).

### 3.3 Prognostic factors for patients with elderly metastatic NSCLC

For the training cohort, 12 variables were considered as independent prognostic factors based on univariate and multivariate Cox proportional hazards models. Our research found that age, gender, race, pathology, grade, tumor site, T stage N stage, surgery, radiotherapy, chemotherapy, and metastatic site had strong correlations with LCSS of elderly patients with metastatic NSCLC (Table 2). Among them, the age at first diagnosis (≥80 years) (HR = 1.121, *p* < 0.001), grade IV (HR = 1.532, *p* < 0.001), T4 stage (HR = 1.380, *p* < 0.001), N3 stage (HR = 1.347, *p* < 0.001), MOM (HR = 1.940, *p* < 0.001), no surgery (HR = 1.725, *p* < 0.001), no radiotherapy (HR = 1.129, *p* < 0.001), no chemotherapy (HR = 2.176, *p* < 0.001) underwent increased risk of death compared with the references, which were similar to the outcomes observed in the multivariate analysis of OS (Table 3).

**TABLE 2:** Univariate and multivariate analysis of LCSS in training cohort

Characteristics	Univariate analysis		Multivariate analysis	
	HR (95% CI)	*p* value	HR (95% CI)	*p* value
**Age**				
60–69	Reference		Reference	
70–79	1.070 (1.021–1.122)	0.005	1.045 (0.996–1.097)	0.070
≥80	1.311 (1.311–1.235)	<0.001	1.121 (1.053–1.193)	<0.001
**Sex**				
Female				
Male	1.123 (1.075–1.173)	<0.001	1.076 (1.028–1.127)	0.002
**Race**				
Black	Reference		Reference	
White	1.026 (0.962–1.094)	0.434	1.126 (1.055–1.202)	<0.001
Other	0.834 (0.754–0.923)	<0.001	0.913 (0.824–1.012)	0.082
**Marital status**				
Married	Reference		Reference	
Unmarried	1.125 (1.077–1.175)	<0.001	1.041 (0.994–1.1090)	0.085
Unknown	1.095 (0.984–1.220)	0.097	1.062 (0.953–1.184)	0.274
**Pathology**				
Adenocarcinoma	Reference		Reference	
Squamous cell carcinoma	1.537 (1.426–1.656)	<0.001	1.282 (1.184–1.387)	<0.001
Others	1.596 (1.477–1.725)	<0.001	1.279 (1.178–1.389)	<0.001
**Grade**				
I	Reference		Reference	
II	1.368 (1.161–1.612)	0.001	1.091 (0.923–1.291)	0.308
III	1.727 (1.475–2.023)	<0.001	1.243 (1.055–1.465)	0.009
IV	2.043 (1.656–2.520)	<0.001	1.532 (1.235–1.901)	<0.001
Unknown	1.696 (1.450–1.984)	<0.001	1.227 (1.044–1.444)	0.013
**Primary location**				
Upper lobe, lung	Reference		Reference	
Middle lobe, lung	0.935 (0.835–1.047)	0.248	0.964 (0.861–1.080)	0.526
Lower lobe, lung	1.016 (0.968–1.067)	0.523	1.054 (1.003–1.107)	0.037
Main bronchus	1.218 (1.107–1.340)	<0.001	1.195 (1.086–1.316)	<0.001
Overlapping lesion of lung	1.275 (1.032–1.575)	0.024	1.374 (1.112–1.698)	0.003
Lung, NOS	1.105 (1.017–1.202)	0.019	1.114 (1.024–1.212)	0.012
**Laterality**				
Right	Reference			
Left	1.008 (0.965–1.052)	0.729		
**T stage**				
T1	Reference		Reference	
T2	1.277 (1.172–1.392)	<0.001	1.261 (1.156–1.375)	<0.001
T3	1.306 (1.202–1.419)	<0.001	1.360 (1.249–1.482)	<0.001
T4	1.348 (1.238–1.468)	<0.001	1.380 (1.265–1.505)	<0.001
**N stage**				
N0	Reference		Reference	
N1	1.160 (1.069–1.259)	<0.001	1.201 (1.106–1.304)	<0.001
N2	1.344 (1.274–1.417)	<0.001	1.333 (1.263–1.408)	<0.001
N3	1.255 (1.176–1.340)	<0.001	1.347 (1.259–1.441)	<0.001
**Surgery**				
Yes				
No/unknown	2.108 (1.869–2.376)	<0.001	1.725 (1.518–1.960)	<0.001
**Radiation**				
Yes				
No	1.298 (1.209–1.394)	<0.001	1.129 (1.045–1.219)	<0.001
**Chemotherapy**				
Yes				
No/unknown	2.005 (1.920–2.093)	<0.001	2.176 (2.078–2.278)	<0.001
**Metastasis site**				
Lung	Reference		Reference	
Liver	1.468 (1.342–1.605)	<0.001	1.512 (1.380–1.656)	<0.001
Bone	1.436 (1.436–1.530)	<0.001	1.561 (1.462–1.668)	<0.001
Brain	1.372 (1.372–1.474)	<0.001	1.575 (1.458–1.701)	<0.001
Multiple	1.855 (1.855–1.969)	<0.001	1.940 (1.823–2.065)	<0.001

Abbreviations: CI, confidence interval; HR, hazard ratio; LCSS, lung cancer‐specific survival.

**TABLE 3:** Univariate and multivariate analysis of OS in training cohort

Characteristics	Univariate analysis		Multivariate analysis	
	HR (95% CI)	P value	HR (95% CI)	P value
**Age**				
60–69	Reference		Reference	
70–79	1.094 (1.045–1.145)	<0.001	1.064 (1.015–1.115)	0.010
≥80	1.309 (1.235–1.388)	<0.001	1.107 (1.041–1.177)	0.001
**Sex**				
Female				
Male	1.126 (1.079–1.175)	<0.001	1.081 (1.033–1.130)	0.001
**Race**				
Black	Reference		Reference	
White	1.014 (0.953–1.080)	0.654	1.116 (1.047–1.189)	0.001
Other	0.813 (0.736–0.897)	<0.001	0.895 (0.809–0.990)	0.030
**Marital status**				
Married	Reference		Reference	
Unmarried	1.136 (1.089–1.185)	<0.001	1.050 (1.004–1.1098)	0.035
Unknown	1.095 (0.996–1.228)	0.061	1.071 (0.963–1.190)	0.205
**Pathology**				
Adenocarcinoma	Reference		Reference	
Squamous cell carcinoma	1.562 (1.452–1.681)	<0.001	1.306 (1.209–1.411)	<0.001
Others	1.600 (1.483–1.727)	<0.001	1.293 (1.193–1.402)	<0.001
**Grade**				
I	Reference		Reference	
II	1.317 (1.125–1.541)	0.001	1.091 (0.893–1.232)	0.558
III	1.658 (1.426–1.928)	<0.001	1.243 (1.0231.401)	0.025
IV	1.947 (1.590–2.384)	<0.001	1.532 (1.193–1.810)	<0.001
Unknown	1.620 (1.395–1.881)	<0.001	1.227 (1.009–1.376)	0.038
**Primary location**				
Upper lobe, lung	Reference		Reference	
Middle lobe, lung	0.939 (0.841–1.049)	0.264	0.968 (0.866–1.081)	0.561
Lower lobe, lung	1.019 (0.972–1.069)	0.439	1.055 (1.006–1.107)	0.027
Main bronchus	1.217 (1.108–1.336)	<0.001	1.198 (1.091–1.316)	<0.001
Overlapping lesion of lung	1.274 (1.036–1.566)	0.022	1.380 (1.122–1.697)	0.002
Lung, NOS	1.100 (1.013–1.194)	0.023	1.109 (1.021–1.205)	0.014
**Laterality**				
Right	Reference			
Left	0.992 (0.952–1.035)	0.712		
**T stage**				
T1	Reference		Reference	
T2	1.250 (1.150–1.359)	<0.001	1.232 (1.132–1.340)	<0.001
T3	1.224 (1.224–1.445)	<0.001	1.325 (1.219–1.440)	<0.001
T4	1.280 (1.180–1.387)	<0.001	1.356 (1.246–1.474)	<0.001
**N stage**				
N0	Reference		Reference	
N1	1.155 (1.067–1.251)	<0.001	1.203 (1.110–1.303)	<0.001
N2	1.331 (1.264–1.402)	<0.001	1.327 (1.258–1.400)	<0.001
N3	1.238 (1.161–1.320)	<0.001	1.335 (1.250–1.426)	<0.001
**Surgery**				
Yes				
No/unknown	2.068 (1.842–2.321)	<0.001	1.698 (1.501–1.921)	<0.001
**Radiation**				
Yes				
No	1.317 (1.228–1.413)	<0.001	1.140 (1.057–1.229)	0.001
**Chemotherapy**				
Yes				
No/unknown	2.035 (1.951–2.123)	<0.001	2.201 (2.104–2.302)	<0.001
**Metastasis site**				
Lung	Reference		Reference	
Liver	1.417 (1.298–1.547)	<0.001	1.459 (1.334–1.595)	<0.001
Bone	1.407 (1.323–1.497)	<0.001	1.532 (1.437–1.633)	<0.001
Brain	1.330 (1.240–1.426)	<0.001	1.529 (1.418–1.649)	<0.001
Multiple	1.793 (1.691–1.901)	<0.001	1.884 (1.774–2.002)	<0.001

Abbreviations: CI, confidence interval; HR, hazard ratio; OS, overall survival.

### 3.4 Calibration and validation of the nomograms

**FIGURE 3:**
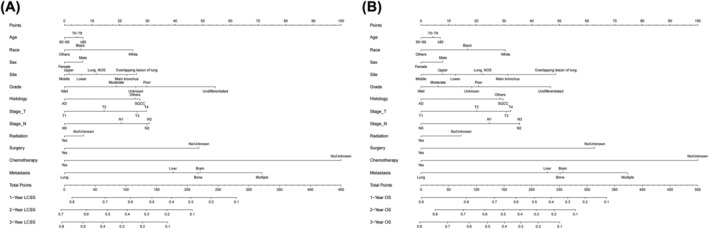
Prognostic nomograms for predicting 1‐, 2‐, and 3‐year lung cancer‐specific survival (LCSS) and overall survival (OS) rate in elderly patients with metastatic NSCLC. (A) LCSS rate; (B) OS rate

**FIGURE 4:**
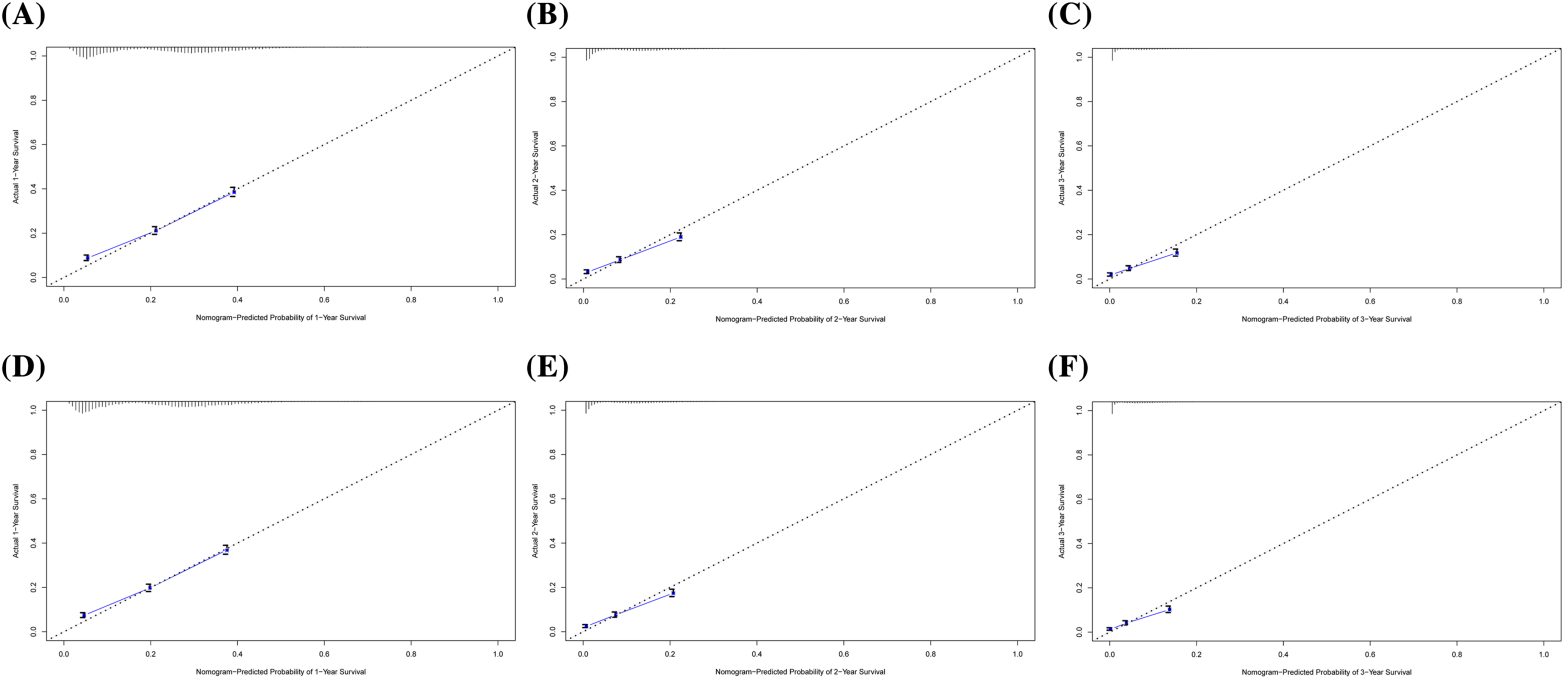
Calibration curves in the training cohort of the nomograms for predicting 1‐, 2‐, and 3‐year LCSS (A–C) and OS (D–F). LCSS, lung cancer‐specific survival; OS, overall survival

Nomograms were developed based on independent prognostic factors identified by multivariate Cox regression analysis to predict 1‐, 2‐ and 3‐year LCSS and OS (Figure 3). The results indicated that the two factors, metastatic site and chemotherapy, had the widest scope of risk scores, indicating the most significant impact on prognosis. For the training set, the C‐index values of nomograms were 0.712 (95% CI: 0.704–0.720) for LCSS (Figure 4A–C) and 0.713 (95% CI: 0.705–0.721) for OS (Figure 4D–F). At the same time, in the validation set, the C‐index values were 0.707 (95% CI: 0.701–0.725) for LCSS (Figure S1A–C) and 0.710 (95% CI: 0.698–0.722) for OS (Figure S1D–F). All had promising predictive value. Moreover, calibration curves showed excellent concordance between actual results and survival rates predicted by the nomograms.

### 3.5 Comparison between nomograms

**FIGURE 5:**
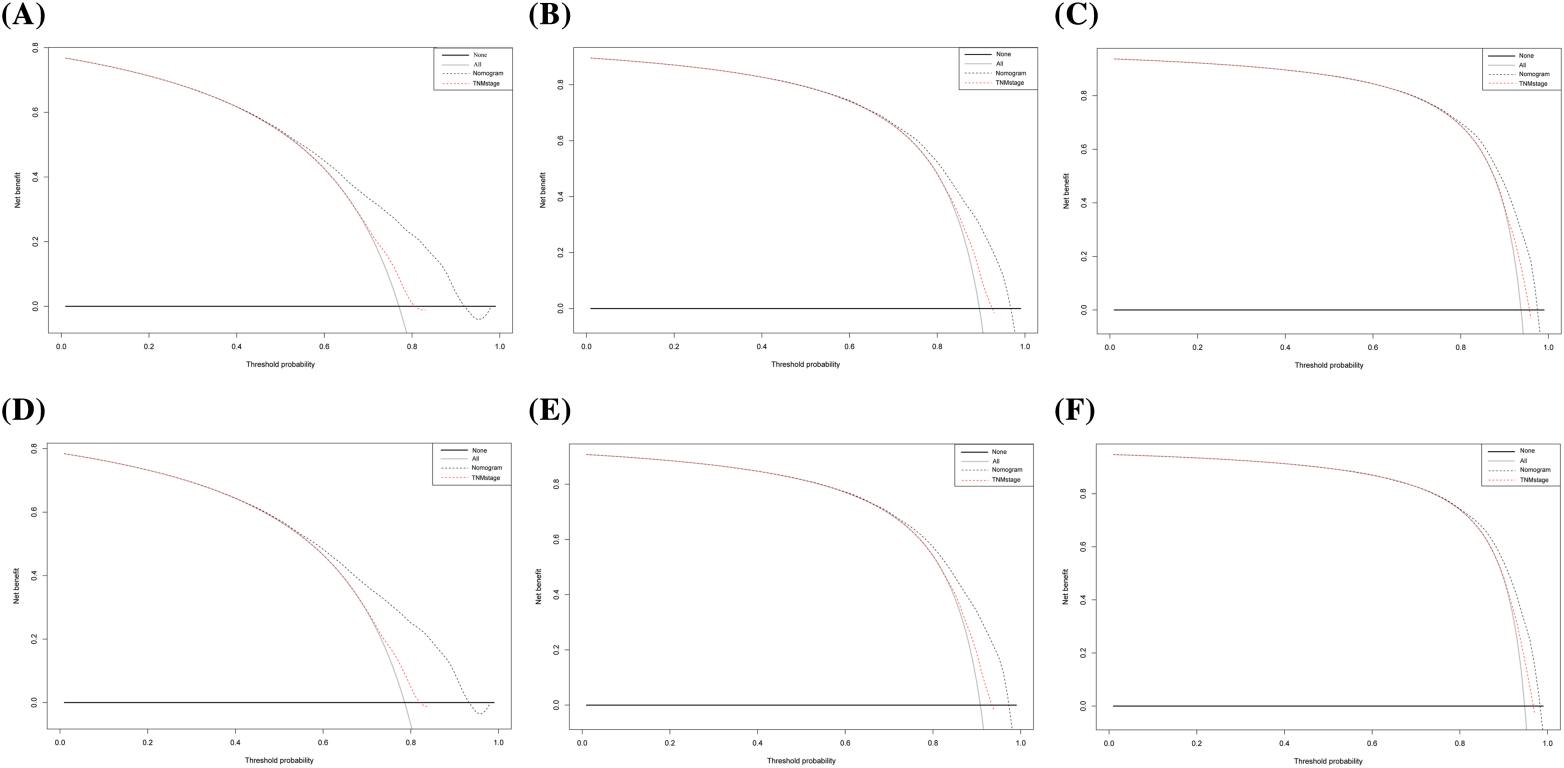
Decision curve analysis in the training cohort of the nomograms and 7th edition AJCC‐TNM staging system for predicting 1‐, 2‐, and 3‐year LCSS (A–C) and OS (D–F). LCSS, lung cancer‐specific survival; OS, overall survival

In the training cohort, the C‐index values for LCSS and OS of the TNM‐staging system were 0.534 (95% CI: 0.524–0.544) and 0.531 (95% CI: 0.523–0.539), respectively, which were considerably lower than the nomograms integrating all independent prognostic variables. Meanwhile, the C‐index of this research in the validation cohort was also remarkably higher than that of the TNM‐staging system, with 0.528 (95% CI: 0.514–0.562) both in LCSS and OS (Table 4). In addition, compared with the TNM staging model, the DCA curves showed excellent net benefit of the novel nomograms in predicting 1‐, 2‐, and 3‐year LCSSS (Figures 5A–C and S2A–C) and OS (Figures 5D–F and S2D–F).

**TABLE 4:** Comparison of C‐indexes between the nomograms and TNM staging system

Characteristics	Training cohort			Validation cohort		
	HR	95% CI	*p* value	HR	95% CI	*p* value
LCSS						
Nomogram	0.712	0.704–0.720		0.707	0.701–0.725	
TNM stage	0.534	0.524–0.544	<0.001	0.528	0.514–0.562	<0.001
OS						
Nomogram	0.713	0.705–0.721		0.710	0.698–0.722	
TNM stage	0.531	0.523–0.539	<0.001	0.528	0.514–0.562	<0.001

Abbreviations: CI, confidence interval; HR, hazard ratio.

## 4 DISCUSSION

We extracted clinical and survival information of 9584 elderly patients with metastatic NSCLC from the SEER database. Twelve risk factors for predicting 1‐, 2‐ and 3‐year LCSS and OS were identified by univariate and multivariable Cox regression models and were used to establish prognostic nomograms. In this research, we firstly used independent demographic and clinicopathologic prognostic factors developed more comprehensive prognostic models for better predicting prognosis of elderly patients with metastatic NSCLC and help clinicians determine individualized treatment strategies.

The population of aging adults in Canada is reported to more than double between 2005 and 2036. The number of patients with advanced NSCLC ≥70 years is increasing, posing unique challenges for treatment decisions.^20^ On the one hand, young and old patients experience different physiological changes related to comorbidities, immune status and nutritional status. On the other hand, elderly patients have reduced renal and hepatic reserve function so as to the potential for drug interactions and treatment‐related toxicity is increased. Combining these causes, age should be a valuable indicator for treatment consideration. Generally, the TNM staging system plays an essential role in the prognosis and treatment decisions of NSCLC patients. Nevertheless, it ignores a variety of important risk factors including race, age, distant metastatic sites as well as other possible markers. What is surprising is that nomogram shows great utility in predicting the probability of clinical events using individual variables, and has become a common prognostic tool in oncology.

In this study, the nomograms incorporated 12 variables: age, gender, race, tumor site, grade, pathology, T stage, N stage, surgery, radiotherapy, chemotherapy, and metastatic site. Meanwhile, chemotherapy as well as distant metastatic sites were the two strongest prognostic predictors. In this study, patients were more inclined to MOM. In other words, older patients were more likely to develop MOM once they experienced distant metastases. This may be for the reason that elderly patients have a tumor microenvironment that favors fibroblast‐mediated angiogenesis and stromal remodelling.^21^, ^22^ In addition, the structure and function of the human immune system change with age. Patients of advanced age are prone to immune senescence, which allows tumors to evade immune system surveillance.^23^, ^24^ Owonikoko et al. investigated NSCLC patients ≥70 years based SEER database and discovered that the patients were predominantly white male,^3^ which was similar to our research. Additionally, the study also made the observation that patients with stage T4 and grade III had a larger proportion of the corresponding variables. This was because the patients included were in advanced tumor stage, and therefore tended to have larger tumor volume along with worse grade. This was the same as the result of Liang's study.^25^

The previous studies reported that patients with NSCLC diagnosed at the age over 80 years contributed to worse LCSS and OS,^26^, ^27^ which were consist with our investigation. Worse nutritional status, reduced physiological reserve, basic diseases and poor tolerance to cancer treatment in the older group might be explanations.^28^, ^29^ Approximately 30%–40% of patients with NSCLC develop metastases at initial diagnosis.^30^ Nevertheless, there is still controversy regarding the influences of metastatic site of lung cancer on prognosis. In this research, multiple Cox analysis demonstrated that patients with MOM with worst prognosis, which were consistent with previous findings.^26^, ^31^ This may be related to the limited effective treatment for MOM. Meantime, the same with previous literature,^27^, ^32^ our data showed that patients received surgery, chemotherapy or radiation therapy earned higher survival rates, indicated that despite elderly patients have reduced physical function, positive treatment could still provide survival benefits if the body could tolerate it. With further research, the association between gender and lung cancer prognosis is being increasingly reported.^33^, ^34^ For example, Barquín et al. found that female with NSCLC experienced significantly longer survival than male(*p* < 0.001).^34^ Our findings also supported this conclusion. One of the hypotheses regarding the better survival outcome exhibited by female probably was associated with different levels of hormone and receptor expression.^35^, ^36^, ^37^ Moreover, several studies reported that lower grade tissue differentiation, lymph node metastasis together with larger tumor size were significantly associated with increased mortality in NSCLC. The same results were well supported by our statistical analysis.

In the end, we verified the performance of the models. The results demonstrated that the C‐index as well as calibration curve of the prediction models performed well in both the training and validation cohorts, indicating that the nomograms had good predictive accuracy and reliability. Additionally, the DCA curves demonstrated that the novel nomograms had higher net benefit and clinical application than TNM staging system.

Altogether, we firstly developed visual prognostic assessment models for elderly patients with metastatic NSCLC. The use of nomogram scores to quantify the survival risk of a patient with organ‐specific metastases to guide clinical treatment and prognostic assessment is a novel concept.

Despite above merits, there were still some limitations in this research. Firstly, some factors affecting prognosis were not included in the SEER database, such as smoking history, family history of cancer, gene mutations and physical state (PS) assessment. Secondly, as essential treatment approaches for NSCLC, the absence of targeted therapy and immunotherapy information from the SEER database was a major restriction of the current study. Moreover, patients with incomplete survival data or clinical details were not included in our research, which might lead to selection bias. Finally, although both internal and external validation sets are proposed to validate the nomogram, in the current study only internal validation was specified. Additional validation studies in independent populations are needed to verify the generalizability of these results before clinical application. Nonetheless, this database provided valuable data for analyzing patterns of elderly patients with metastatic NSCLC across the United States.

## 5 CONCLUSION

To our best knowledge, this was the first large‐scale population‐based research with nomograms to explore the prognosis of elderly patients with metastatic NSCLC.

All patients were followed up in detail. The novel models had excellent predictive performance and can intuitively predict patient survival. Meanwhile, the nomograms could be used as effective tools to assist clinicians in guiding individualized treatment decisions and consequently reduced the medical burden to some extent.

## CONFLICT OF INTERESTS

The authors have no conflict of interests to declare.

## ETHICS STATEMENT

As all data were obtained from the SEER database, informed patient consent and ethical approval were not required.

## AUTHOR CONTRIBUTIONS

HSS and CW designed the study. HSS collected the data, analyzed the data, and finalized the manuscript. XYY and YHR participated in the collection and assembly of data. ML, HPD and CW revised the manuscript. All authors contributed to the article and approved the final version of manuscript.

## Supporting information


**Figure S1.** Calibration curves in the validation cohort of the nomograms for predicting 1‐, 2‐, and 3‐year LCSS (A–C) and OS (D–F). LCSS, lung cancer‐specific survival; OS, overall survival.

**Figure S2.** Decision curve analysis in the validation cohort of the nomograms and 7th edition AJCC‐TNM staging system for predicting 1‐, 2‐, and 3‐year LCSS (A–C) and OS (D–F). LCSS, lung cancer‐specific survival; OS, overall survival.

## Data Availability

The data that support the findings of this study are available from the corresponding author upon reasonable request.
