# Supplementary figures and images for: Construction and validation of prognostic nomograms for elderly patients with metastatic non‐small cell lung cancer
Source: Clin Respir J. 2022 May 5;16(5):380–93. doi: 10.1111/crj.13491 (PMC9366578; doi:10.1111/crj.13491)

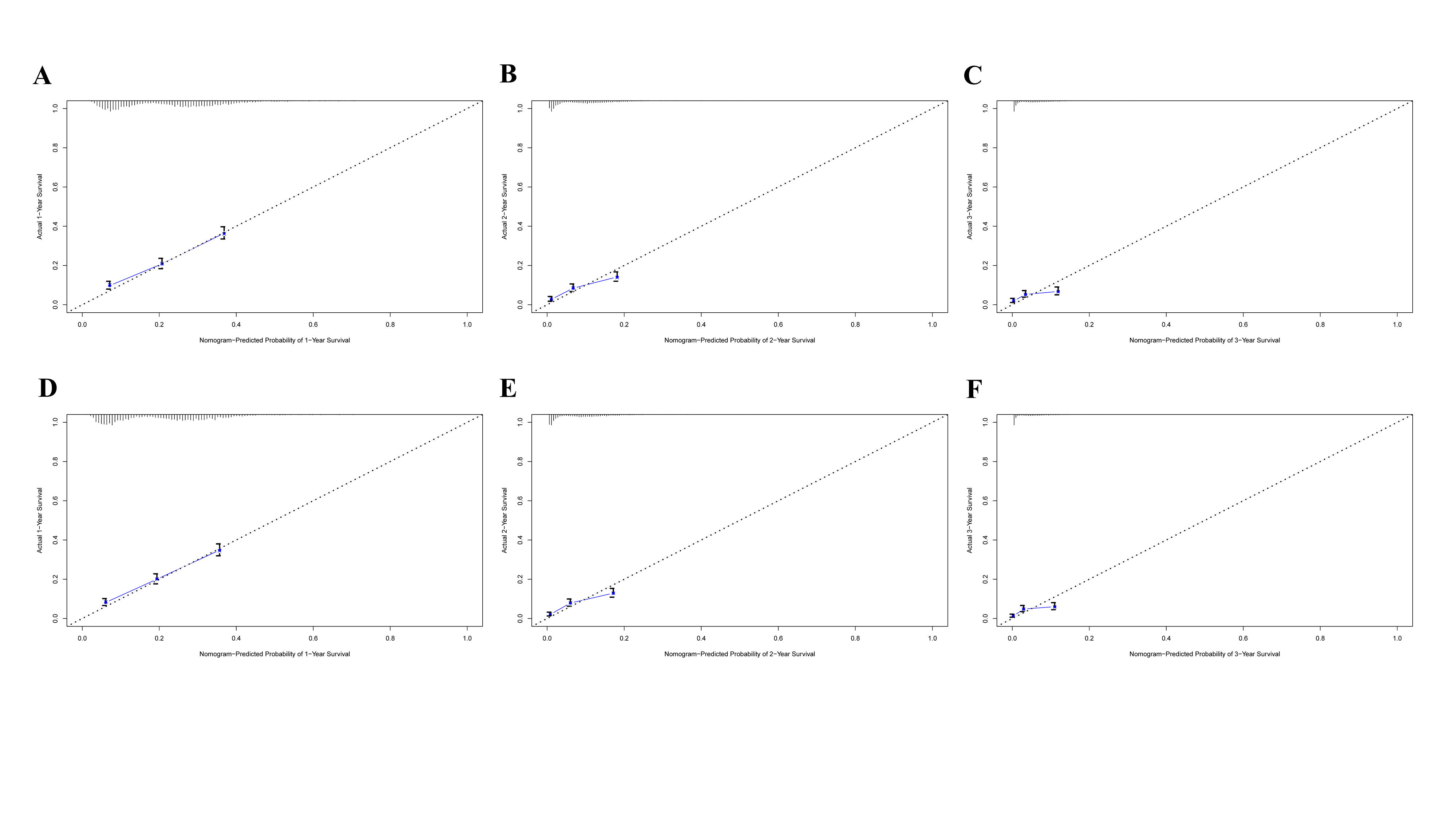

Supplement: Supplementary file 1 — Figure S1. Calibration curves in the validation cohort of the nomograms for predicting 1‐, 2‐, and 3‐year LCSS (A–C) and OS (D–F). LCSS, lung cancer‐specific survival; OS, overall survival. [file CRJ-16-380-s001.TIF]

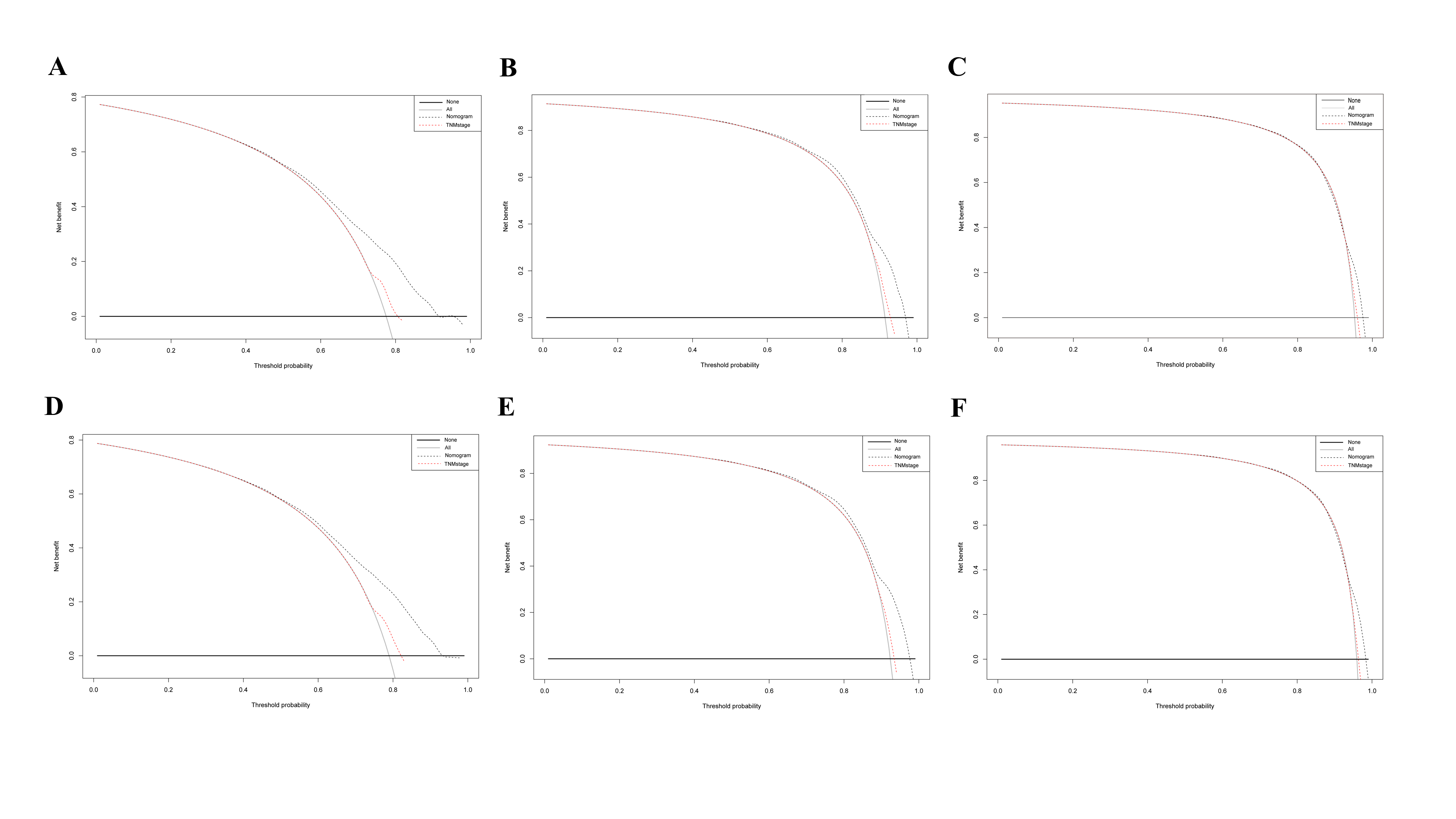

Supplement: Supplementary file 2 — Figure S2. Decision curve analysis in the validation cohort of the nomograms and 7th edition AJCC‐TNM staging system for predicting 1‐, 2‐, and 3‐year LCSS (A–C) and OS (D–F). LCSS, lung cancer‐specific survival; OS, overall survival. [file CRJ-16-380-s002.TIF]
